# A remarkable and durable response to tislelizumab treatment of an anaplastic thyroid carcinoma without targetable genomic alterations: a case report

**DOI:** 10.3389/fimmu.2025.1544604

**Published:** 2025-02-12

**Authors:** Jingjing Chai, Jiaqi Lv, Jian Xiong, Xiuwen Chen, Senyuan Luo, Zhiguo Luo, Ming Luo

**Affiliations:** ^1^ Department of Oncology, Taihe Hospital, Hubei University of Medicine, Shiyan, Hubei, China; ^2^ Cardiovascular Department, Taihe Hospital, Hubei University of Medicine, Shiyan, Hubei, China; ^3^ Department of Pathology, Taihe Hospital, Hubei University of Medicine, Shiyan, Hubei, China; ^4^ Key Laboratory of Cancer Therapy Resistance and Clinical Translational Study, Shiyan, Hubei, China

**Keywords:** anaplastic thyroid carcinoma, PD-1 inhibitor, PD-L1 expression level, whole exome sequencing, case report

## Abstract

Anaplastic thyroid carcinoma (ATC) is a rare and highly aggressive malignancy characterized by a poor prognosis, with a median survival time of approximately 3 to 4 months. In this report, we present a case involving a 59-year-old patient diagnosed with ATC, who experienced swift local recurrence and pulmonary metastasis following radical thyroidectomy. Comprehensive Sanger sequencing of the resected tumor tissue revealed no mutations in the TERT promoter or the BRAF V600E gene. The patient exhibited rapid recurrence post-surgery and was deemed unsuitable for immediate surgical intervention. The patient was unable to tolerate chemotherapy; therefore, radiotherapy was administered initially to prevent airway compression resulting from disease progression. During the course of radiotherapy, pulmonary metastasis developed, yet the patient remained intolerant to both chemotherapy and anti-angiogenic therapy. Immunohistochemical analysis revealed a high expression of PD-L1. Whole exome sequencing (WES) indicated a tumor mutation burden (TMB) of 2.98 mut/Mb, microsatellite stability (MSS), and identified 10 missense mutations, 1 nonsense mutation, and 1 frameshift insertion. However, none of these mutations have corresponding targeted therapies. Consequently, we administered tislelizumab as an immunotherapeutic intervention. The patient exhibited significant regression in cervical metastatic lymph nodes and pulmonary metastatic tumors, achieving a sustained remission lasting 14 months, culminating in complete remission, without experiencing any adverse events related to immune checkpoint inhibitors. This case demonstrates the efficacy of second-line monotherapy with an immune checkpoint inhibitor (ICI) for ATC in a patient unable to tolerate chemotherapy and anti-angiogenic anlotinib treatment, thereby offering a viable treatment strategy for ATC patients.

## Introduction

Anaplastic thyroid carcinoma (ATC) is an uncommon and aggressive malignant tumor, representing 1% to 2% of all thyroid cancers. Notably, over 90% of patients succumb to the disease within six months following diagnosis. ATC is characterized by its high degree of malignancy, rapid progression, and invasive nature. At the time of detection, most patients present with a substantial local mass, rapid tumor growth, and pronounced clinical manifestations related to compression or infiltration. Additionally, distant metastasis is often observed (1, 2). Among thyroid cancers, ATC exhibits the highest malignant potential and is typically diagnosed at an advanced stage (stage IV), with limited effective treatment options available. Through comprehensive research into the molecular biology of thyroid cancer, there has been an increased utilization of targeted therapies and immune checkpoint inhibitors (ICIs) in the management of ATC. In May 2018, the Food and Drug Administration (FDA) sanctioned the use of a combination of dabrafenib and trametinib for the treatment of advanced ATC with BRAF V600E mutations. Subsequently, in 2020, the FDA approved pembrolizumab for the treatment of tumors characterized by a high tumor mutation burden (>10 mut/Mb), as well as recurrent, refractory, or metastatic solid tumors. Nonetheless, it is important to note that, to date, no ICIs have received FDA approval specifically for the treatment of ATC. The “2021 Anaplastic Thyroid Carcinoma Management Guidelines” issued by the American Thyroid Association indicate that for patients with stage IVC ATC exhibiting high PD-L1 expression, ICIs may be employed as either first-line or subsequent therapeutic options in the absence of other targetable mutations (3). In this report, we present a case involving a patient with advanced ATC who experienced rapid recurrence and pulmonary metastasis following surgical intervention. The patient achieved complete remission (CR) through monotherapy with the immune checkpoint inhibitor tislelizumab. Additionally, we conducted an analysis of the patient’s gene mutations utilizing whole exome sequencing (WES). This report is the initial one to investigate and validate the efficacy of monotherapy using the ICI tislelizumab for the rapid postoperative metastasis of ATC. This case report was written in accordance with the CARE guidelines to ensure completeness and transparency.

## Case presentation

**Figure 1:**
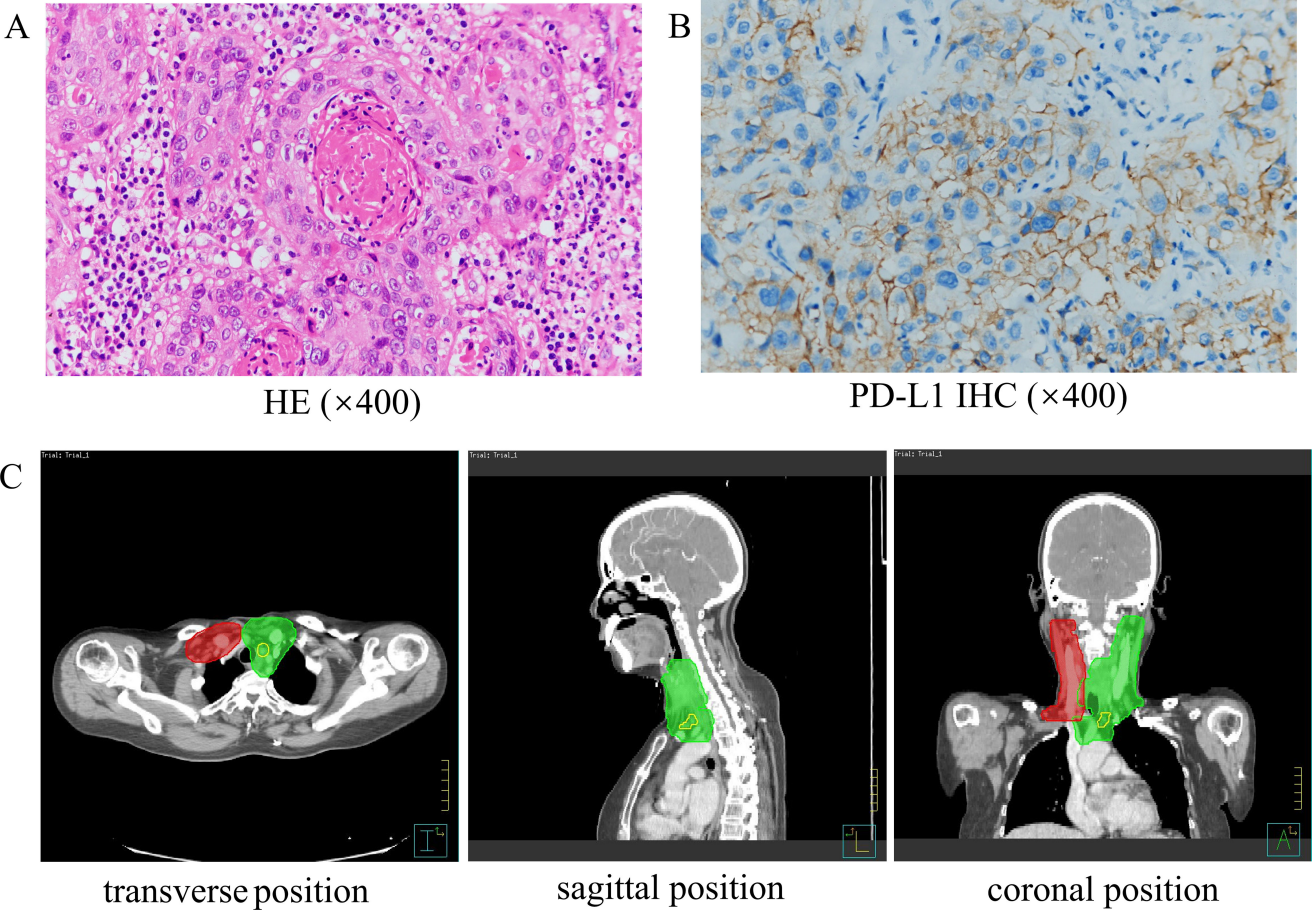
The pathology and radiotherapy target volume of the patient. **(A)** HE staining of the primary tumor (×400). **(B)** PD-L1 immunohistochemistry (×400). **(C)** The map of patient’s radiotherapy target volume. The dose was 6600 cGy in the yellow part (GTVnd), 6000 cGy in the green part (CTV1: high-risk regions) and 5400 cGy in the red part (CTV2: low-risk regions).

**Figure 2:**
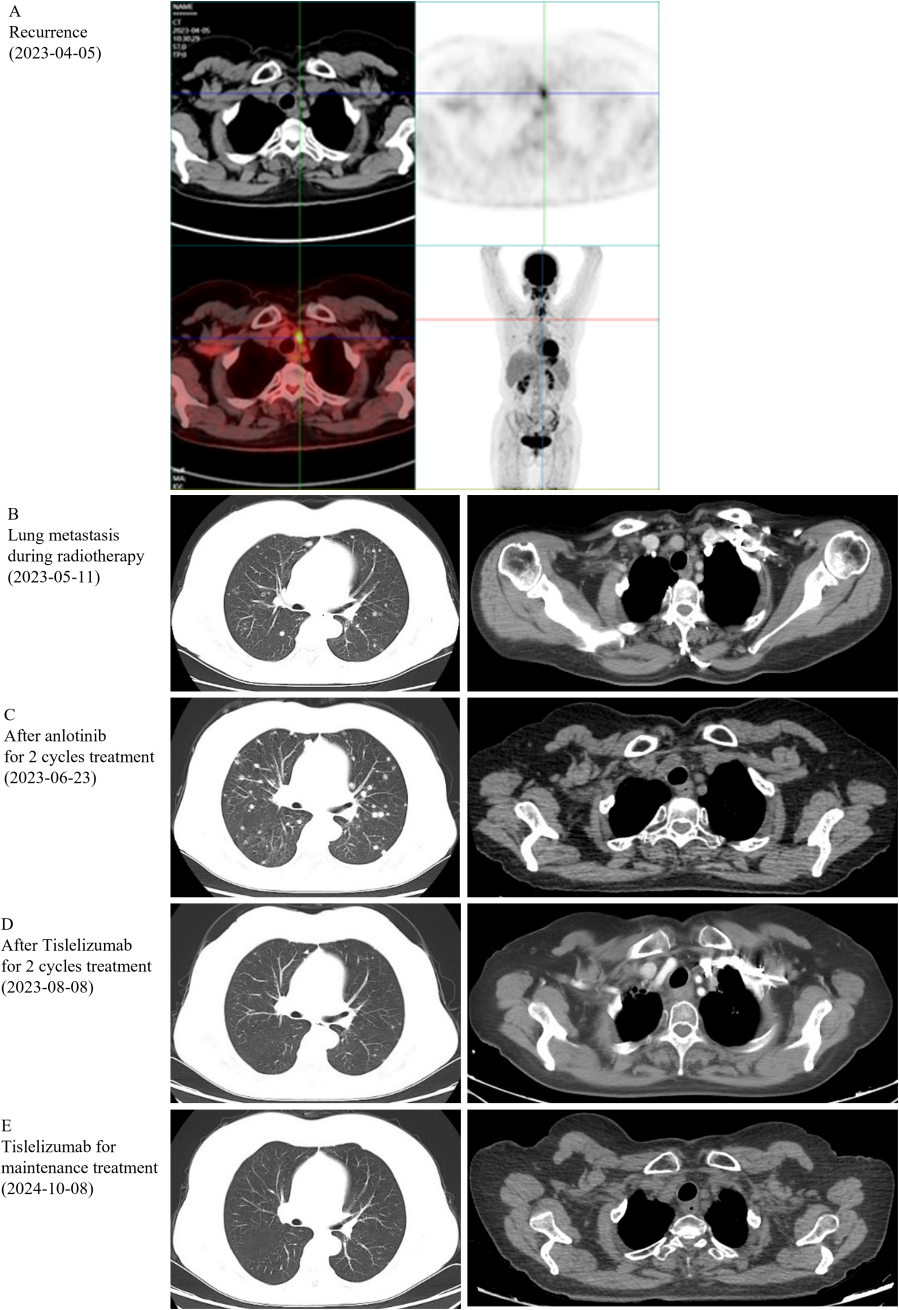
Imaging results. **(A)** PET-CT was performed on the recurrence, on Apr 5, 2023. **(B-E)** Computerized tomography (CT) was performed after different stages of treatment.

**Figure 3:**
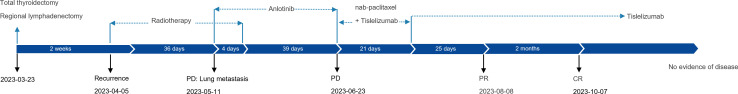
Case summary. Summary of disease development and treatment. CR, complete response; PD, progressive disease; PR, partial response.

On March 3, 2023, a 59-year-old female patient presented with a rapidly enlarging thyroid mass, accompanied by pain and dysphagia, without respiratory symptoms. A color Doppler ultrasound examination revealed multiple hypoechoic nodules within the bilateral thyroid lobes, with the largest nodule located in the mid-region of the left lobe, measuring approximately 47×30×27 mm (TI-RADS 3). Subsequent neck computed tomography (CT) imaging demonstrated an enlargement of the left thyroid lobe, characterized by a low-density mass with heterogeneous density and several peripheral hyperdense areas, with the largest dimension measuring approximately 3.1×3.9×5.0 cm, and the trachea was compressed and shifted to the right side. The bilateral cervical and submandibular lymph nodes exhibited slight enlargement, with the largest measuring approximately 0.7 cm in short-axis diameter. On March 23, 2023, the patient underwent a total thyroidectomy accompanied by regional lymph node dissection. The postoperative pathological analysis revealed the presence of squamous cell carcinoma in the left thyroid lobe and isthmus (**Figure 1A**), classified as a subtype of ATC according to the 2022 WHO classification of thyroid tumors. The tumor measured 5×3.5×2.5 cm and exhibited a mixed solid and cystic composition, with evidence of nerve and vascular invasion, as well as invasion of the thyroid capsule. One central lymph node showed metastasis of cancer cells. However, all surgical margins were negative. Immunohistochemistry results showed TTF-1 (a few weak +), PAX8 (most +), P40 (+), P63 (+), CD56 (-), Ki-67 (50% +), P16 (-), P53 (-), CD117 (-), CD5 (-), CK5/6 (+). Sanger sequencing analysis revealed the absence of C228T and C250T mutations in the promoter region of the TERT gene. Additionally, no mutations were identified in exon 15 of the BRAF gene. Immunohistochemical analysis of programmed death-ligand 1 (PD-L1) demonstrated a Combined Positive Score (CPS) of approximately 60 (**Figure 1B**). On April 5, 2023, two weeks post-operatively, a PET-CT scan was conducted, which indicated post-thyroidectomy changes with increased local metabolic activity (SUV max 6.3), warranting further observation. The scan also revealed scattered lymph nodes in the lower neck (region VI) exhibiting elevated metabolic activity, suggestive of potential metastasis (**Figure 2A**). The patient was unable to tolerate surgery and chemotherapy, consequently, radiotherapy was administered to the tumor bed, metastatic lymph nodes, and lymphatic drainage area. The treatment plan included a dose of 60 Gy/2 Gy/30 fractions for high-risk region, a dose of 54 Gy/1.8 Gy/30 fractions for low-risk region, and a dose of 66 Gy/2.2 Gy/30 fractions for the metastatic lymph node in the neck region VI (**Figure 1C**). On the 17th fraction of radiotherapy, the patient acutely developed symptoms of chest tightness, dyspnea, and respiratory distress. A neck CT scan revealed no new lesions, while a chest CT scan indicated the presence of multiple small nodules in both lungs, some of which appeared to be new and were suspected to be metastatic (**Figure 2B**). Owing to the development of radiation-induced oral mucositis, decreased food intake, significant weight loss, and an Eastern Cooperative Oncology Group (ECOG) performance status score of 3, the patient was temporarily unable to tolerate chemotherapy. On May 15, 2023, treatment with anlotinib was initiated; however, the patient experienced hemoptysis following two cycles of therapy. A subsequent chest CT scan on June 23, 2023, demonstrated multiple nodules in both lungs, with some nodules being newly identified and suspected to be metastatic (**Figure 2C**). Given increased food intake and improved nutritional status, the patient achieved an ECOG performance status of 1 and the patient’s elevated PD-L1 expression, a treatment regimen comprising immunotherapy (tislelizumab 200 mg every three weeks) in conjunction with chemotherapy (albumin-bound paclitaxel 125 mg/m² on days 1 and 8, every three weeks) was initiated on June 28, 2023. However, following the initial day of chemotherapy, the patient developed grade 3 bone marrow suppression and joint pain, rendering them unable to tolerate the chemotherapy. Consequently, the treatment was adjusted to continue with immunotherapy alone. Following two cycles of immunotherapy, a review of the chest CT scan indicated postoperative improvement in the thyroid malignant tumor and multiple lung metastases (**Figure 2D**). Subsequently, monotherapy with the immunotherapeutic agent was continued, resulting in sustained remission for 14 months without any adverse reactions (**Figure 2E**). A summary of disease progression and treatment is presented in **Figure 3**.

Upon obtaining the patient’s informed consent, the tumor tissue and peripheral blood samples were submitted to BGI for WES. The germline analysis identified 10,602 synonymous mutations, 10,023 missense mutations, and 259 frameshift mutations. The somatic mutation analysis encompassed somatic single nucleotide variants (SNVs), somatic insertions and deletions (InDels), somatic copy number variations (CNVs), and somatic structural variations (SVs). Specifically, the somatic SNV analysis revealed 10 missense mutations and 1 nonsense mutation, while the somatic InDel analysis identified 1 frameshift insertion (**Table 1**). The tumor mutational burden (TMB) was calculated at 2.98 mut/Mb, and the sample was classified as microsatellite stable (MSS).

**Table 1:** Somatic mutation results of the patient.

Gene name	Variant classification	Variant type	cDNA change	Protein change	Chromosome	Exon number
NRAS	Missense_Mutation	SNP	c.182A>G	p.Q61R	1	3
GON4L	Missense_Mutation	SNP	c.3382T>C	p.S1128P	1	21
EVX2	Missense_Mutation	SNP	c.1219G>A	p.A407T	2	3
TUBB2B	Missense_Mutation	SNP	c.743C>T	p.A248V	6	4
RFX6	Missense_Mutation	SNP	c.450A>C	p.L150F	6	3
AQP2	Missense_Mutation	SNP	c.643G>T	p.G215C	12	4
SAV1	Nonsense_Mutation	SNP	c.823C>T	p.Q275*	14	4
RHCG	Missense_Mutation	SNP	c.880G>T	p.G294C	15	6
USH1G	Missense_Mutation	SNP	c.941C>A	p.T314N	17	2
FSTL3	Missense_Mutation	SNP	c.304G>A	p.V102M	19	3
GRIN2D	Missense_Mutation	SNP	c.3284C>T	p.P1095L	19	13
GTF3C5	Frame_Shift_Ins	INS	c.640_641insA	p.H214fs	9	4

## Discussion

ATC represents the most aggressive and prognostically unfavorable form of thyroid neoplasms. The etiology of ATC remains largely unidentified, although its incidence may be influenced by a combination of environmental, genetic, hormonal, and other factors. Due to its poorly differentiated nature, ATC exhibits minimal iodine uptake and demonstrates resistance to chemotherapy, rendering treatments such as radioactive iodine (RAI) therapy and conventional chemotherapy largely ineffective (4). In recent years, significant advancements have been made in understanding the molecular mechanisms underlying the onset and progression of thyroid cancer. Research has identified that mutations in genes such as paired box gene 8 (PAX8), the tumor suppressor gene TP53, and BRAF V600E may play critical roles in the development of ATC. Presently, the only targeted pharmacological therapy approved for ATC is the combination of dabrafenib and trametinib, specifically for the treatment of advanced cases with BRAF V600E mutations. Multi-target tyrosine kinase inhibitors have been the focus of clinical research concerning ATC. Zheng et al. (5) conducted a clinical trial to assess the efficacy of anlotinib-based chemotherapy in patients with locally advanced or metastatic ATC. Among the 25 patients enrolled in the study, one achieved a CR, while 14 experienced a partial response. The study reported an overall response rate of 60% and a disease control rate of 80%. Numerous studies have been conducted by researchers on PD-1/PD-L1, revealing that a significant proportion of cancer patients derive limited benefit from monotherapy with immune checkpoint inhibitors. This limitation is largely attributed to vascular abnormalities prevalent in most solid tumors, which facilitate immune evasion. Thyroid cancer, in particular, is characterized as a “cold tumor” and typically exhibits a poor response to immune checkpoint inhibitors. Nevertheless, Shipra Agarwal and colleagues undertook the most extensive cohort study of ATC to date, evaluating PD-L1 expression in ATC. Their findings indicate that a majority of ATC cases express PD-L1, thereby suggesting a potential avenue for immunotherapy (6). At present, the majority of clinical investigations conducted on ATC focus on immune combination therapies. These include the combination of cemiplimab with dabrafenib and trametinib (NCT04238624), lenvatinib with pembrolizumab (NCT04171622), and atezolizumab with either chemotherapy or targeted agents (NCT03181100) (7). Spartalizumab (PDR001), a human IgG4 antibody targeting PD-1 developed by Novartis, represents the first immune checkpoint inhibitor to demonstrate efficacy in ATC. A Phase II cohort study revealed that Spartalizumab was administered to patients with locally advanced and/or metastatic ATC, yielding an overall response rate of 19%. Notably, in the subgroup with PD-L1 expression levels of 50% or higher, the response rate increased to 35%, and the 1-year survival rate for patients with positive PD-L1 expression was 52.1%. Importantly, the therapeutic response to Spartalizumab was observed to be independent of the presence of the BRAF V600E mutation (8). In this instance, TMB was measured at 2.98 mut/Mb, indicating a low level of tumor mutational activity. The patient was unable to tolerate anlotinib anti-angiogenic therapy and chemotherapy. Given the positive expression of PD-L1 and the relatively low incidence of adverse reactions associated with ICIs, monotherapy with an immune treatment was administered. This approach led to sustained remission, and the patient is currently in a state of complete remission. It is evident that patients with ATC who exhibit low tumor mutation burden yet high PD-L1 expression may also derive benefit from monotherapy with immunotherapy. This is particularly advantageous for patients who are unable to undergo chemotherapy or targeted therapy, thereby presenting novel therapeutic opportunities. The evaluation of immunotherapy efficacy in thyroid anaplastic carcinoma may initially consider the PD-L1 expression level. The patient did not respond to chemotherapy or antiangiogenic therapy with anlotinib but demonstrated a favorable response to monotherapy with immunotherapy. Considering the patient underwent radiotherapy postoperatively, it was uncertain whether the synergistic antitumor effect of radiotherapy combined with immunotherapy was involved in this case.

Currently, an increasing number of studies have demonstrated that the combination of radiotherapy and immunotherapy exerts synergistic antitumor effects through multiple mechanisms. Radiotherapy can induce immunogenic cell death in tumor cells, thereby releasing tumor antigens that activate the host’s antitumor immune response (9). Additionally, radiotherapy can modulate the tumor microenvironment, shifting it from an immunosuppressive to an immunostimulatory state (10). A case reported by Xing et al. showed significant tumor shrinkage in ATC through the combination of tislelizumab immunotherapy and radiotherapy (11). However, whether the sequence of radiotherapy followed by immunotherapy in this patient achieves synergistic antitumor effects remains to be further investigated.

Furthermore, WES might also offer potential insights into the immunotherapeutic management of these patients. To the best of our knowledge, this is the first instance of WES analysis of ATC, and this information may potentially be beneficial for the fundamental research of immunotherapy. We used WES analysis to obtain more comprehensive genomic data, identifying mutations in RAS, Follistatin-like 3 (FSTL3), and GON4L as potentially significant. Although targeted therapies for these specific genetic mutations are currently unavailable, existing studies suggest potential associations with immunotherapy.

RAS point mutations represent the most prevalent genetic alterations in follicular thyroid carcinoma (FTC), occurring in approximately 40% to 50% of cases. These mutations are also present in undifferentiated carcinoma and refractory thyroid cancer. Several studies have indicated that RAS mutations are predictive of increased invasiveness and poorer prognosis in thyroid cancer (12). NRAS, a member of the RAS gene family, frequently appears as a mutated oncogene in human tumors. Among thyroid cancers, the NRAS Q61R mutation is the most commonly observed, surpassed only by BRAF mutations (13). The NRAS gene encodes the N-Ras protein, which plays a pivotal role in regulating gene transcription activity and the cell cycle via the RAS-RAF-MEK-ERK signaling pathway, a pathway intimately associated with cellular proliferation (14). According to data from the ImmPort data portal (https://www.immport.org/home), which has cataloged 2,483 immune-related genes, the NRAS Q61R mutation is included among these genes. Recent research has identified an association between NRAS mutations and immunotherapy, noting that these mutations can induce antigen-specific T cell responses (15). However, the role of NRAS mutations in the context of immunotherapy remains contentious. In a study conducted by S. Byeon et al., the gene expression profiles of Asian patients with advanced melanoma were analyzed to investigate the correlation between various melanoma subtypes, distinct gene mutations, and the efficacy of immune checkpoint therapy. The findings indicated that NRAS mutations were significantly correlated with resistance to ICI, with statistical significance (P<0.05) (16). A study conducted by the Italian Melanoma Intergroup (IMI) determined that NRAS mutations did not influence the characteristics of primary and metastatic melanoma nor the outcomes of checkpoint inhibitor immunotherapy (17). In contrast, Jaeger et al. performed a systematic review of 16 clinical studies, revealing that melanoma cases harboring NRAS mutations exhibited a higher objective response rate (ORR) to immune checkpoint inhibitors compared to wild-type cases (RR=1.28, 95% CI: 1.01–1.64) (18). The variability in the findings of these studies may be attributed to factors such as racial demographics, disease characteristics, treatment modalities, and the presence of additional mutations. Nonetheless, there is presently a lack of studies assessing the efficacy of the NRAS Q61R mutation in conjunction with immunotherapy in thyroid cancer. Whole exome sequencing of this particular case revealed the presence of the NRAS Q61R mutation and PDL-1 positivity, with the patient demonstrating a favorable response to immune checkpoint inhibitor therapy. The potential of the NRAS Q61R mutation to serve as a predictive biomarker for ATC immunotherapy, as well as its correlation with PD-L1 expression levels and the efficacy of immunotherapy, necessitates further investigation through both basic and clinical research.

FSTL3 is a secreted glycoprotein originating from adipose tissue, the reproductive system, pancreas, liver, skeletal muscle, and placenta. It plays a critical role in regulating glucose-lipid metabolism and is implicated in tumorigenesis and cancer progression (19). Furthermore, FSTL3 is associated with the prognosis of various cancers and the efficacy of immunotherapy. Research conducted by Chao Yang et al. suggests that the overexpression of FSTL3 may contribute to the formation of an inhibitory immune microenvironment by promoting the polarization of macrophages and fibroblasts, as well as the depletion of T-cells. This process facilitates immune escape and enhances sensitivity to immunotherapeutic interventions. In most cancer types, there is a significant correlation between the expression level of FSTL3 and the expression of immune checkpoint molecules (20). In a phase II study investigating the combination of immunotherapy with definitive chemoradiotherapy in esophageal squamous cell carcinoma, GON4L mutations were associated with a reduced incidence of immune-related adverse events (irAEs) (P = 0.036) (21). The patient exhibited a favorable response to long-term use of ICIs without experiencing irAEs; however, further research is needed to confirm whether this outcome is related to FSTL3 expression and GON4L mutations.

Somatic mutations identified in the exonic regions of the patient’s genome contribute to the tumorigenesis of various cancers. However, their association with the prognosis of ATC, the effectiveness of immunotherapy, and the incidence of adverse reactions to immunotherapy remains ambiguous. The observed rapid recurrence with pulmonary metastasis post-surgery, along with the absence of irAEs during prolonged ICI therapy, may be attributable to these genetic alterations. Nonetheless, further research is required to substantiate these potential correlations.

This case report presents ATC limited literature on the use of immune monotherapy with tislelizumab. The detailed clinical and WES data of this case provide valuable insights into the pathophysiology and management of this disease. The successful use of immune monotherapy in this case highlights a potential therapeutic approach that may benefit other patients with similar presentations. Additionally, comprehensive follow-up data offer a detailed understanding of the patient’s response to treatment over time. However, as a single case report, our findings are limited by the small sample size and lack of a control group, precluding definitive conclusions regarding the efficacy and safety of the treatment. Future studies with larger cohorts and longer follow-up periods are needed to validate our findings and further explore the therapeutic potential of monotherapy with immunotherapy in ATC.

## Conclusion

This case report details a patient with ATC who experienced recurrence shortly after undergoing radical surgery. The patient exhibited high PD-L1 expression and an NRAS Q61R mutation, but had a low TMB and was MSS. Due to the patient’s inability to tolerate chemotherapy and the anti-angiogenic inhibitor anlotinib, treatment with the PD-1 inhibitor tislelizumab was initiated, ultimately resulting in complete remission. This report investigates and substantiates the efficacy of immune monotherapy in the management of rapidly progressing ATC with low TMB in later treatment lines, suggesting that this therapeutic approach warrants further investigation in ATC.

## Data Availability

The original contributions presented in the study are included in the article/supplementary material, further inquiries can be directed to the corresponding author/s.
